# Causes and Cure of Diseases of the Feet: With Practical Suggestions as to Their Clothing

**Published:** 1862-08

**Authors:** 


CAUSES AND CURE OF DISEASES OF THE FEET: With Practical Suggestions as to their Clothing. By C. H. CLEVELAND, M.D. Cincinnati: Printed by BRADLEY & WEBB. 1862.

This is an unbound monograph of 111 pages, consisting mainly of the re-publication of articles originally appearing in the *Cincinnati Journal of Rational Medicine.* From a hasty examination, we think it a well written and judicious practical treatise on the diseases of the feet; many of which are troublesome and difficult to cure.

